# MiR-27b-3p promotes migration and invasion in colorectal cancer cells by targeting HOXA10

**DOI:** 10.1042/BSR20191087

**Published:** 2019-12-06

**Authors:** Xiangling Yang, Junxiong Chen, Yao Liao, Lanlan Huang, Chuangyu Wen, Mengmeng Lin, Weiqian Li, Yonglin Zhu, Xiaojian Wu, Aikichi Iwamoto, Zhongyang Wang, Huanliang Liu

**Affiliations:** 1 Guangdong Provincial Key Laboratory of Colorectal and Pelvic Floor Diseases, Guangdong Institute of Gastroenterology, The Sixth Affiliated Hospital, Sun Yat-sen University, Guangzhou, China; 2 Department of Clinical Laboratory, The Sixth Affiliated Hospital, Sun Yat-sen University, Guangzhou, China; 3 Japan Agency for Medical Research and Development (AMED), Tokyo, Japan; 4 Department of Urology, The Sixth Affiliated Hospital, Sun Yat-sen University, Guangzhou, China

**Keywords:** colorectal cancer, HOXA10, invasion, migration, miR-27b-3p

## Abstract

**Purpose:** Dysregulation of microRNAs (miRNAs) contributes to tumor progression via the regulation of the expression of specific oncogenes and tumor suppressor genes. One such example, miR-27b-3p, has reportedly been involved in tumor progression in many types of cancer. The aim of the present study was to delve into the role and the underlying mechanism of miR-27b-3p in colorectal cancer (CRC) cells. **Methods:** In the present study, we detected the expression level of miR-27b-3p by RT-PCR. The effect of miR-27b-3p overexpression on cell proliferation in CRC cells was evaluated by cell counting and Edu assays. Transwell migration and invasion assays were used to examine the effects of cell migration and invasion. Bioinformatics, luciferase reporter assay and western blot assay were performed to identify the target of miR-27b-3p. **Results:** Here, we have demonstrated that although miR-27b-3p can affect cell morphology, it has no observable effect on the proliferation of CRC cells. However, it significantly promotes the migration and invasion of CRC cells. We discovered that HOXA10 was a newly identified target of miR-27b-3p in CRC cells, as confirmed by bioinformatics, western blots and dual luciferase reporter assay. Furthermore, the overexpression of miR-27b-3p or the suppression of HOXA10 can activate the integrin β1 signaling pathway. In conclusion, our results reveal a new function of miR-27b-3p that demonstrates its ability to promote CRC cell migration and invasion by targeting the HOXA10/integrin β1 cell signal axis. **Conclusion:** This may provide a mechanism to explain why miR-27b-3p promotes CRC cell migration and invasion.

## Introduction

Due to significant advancements in early diagnosis and treatment for colorectal cancer (CRC), its incidence and mortality in developed countries have been declining for the past several decades [1]. However, CRC remains one of the most common malignancies worldwide [2]. The main therapeutic obstacles are recurrence and, in advanced CRC, the development of distant metastasis. Accordingly, investigating the molecular mechanisms responsible for CRC progression remains crucial.

microRNAs (miRNAs) are a class of small non-coding RNAs (19–25 bp) that regulate gene function via gene silencing induced by binding to the corresponding RNAs [3]. There is increasing evidence that dysregulation of miRNAs is responsible for the pathogenesis of CRC, and some miRNA may be important for the diagnostic and therapeutic applications in CRC [4]. For instance, a report stated that miR-92 is significantly elevated in the plasma of CRC patients and, hence, may be a potential marker for CRC screening [5]. miR-17-5p has been reported as a key regulator of CRC progression via repressing its target gene P130 and promoting drug resistance by targeting PTEN [6,7]. We have previously reported that miR-181d, whose main functions are to inhibit cancer cell proliferation, migration and invasion by targeting PEAK1, which is down-regulating in CRC patients, suggesting therapeutic applications [8]. miR-19a is associated with lymphatic metastasis and mediates the TNF-α-induced epithelial–mesenchymal transition in colorectal cancer [9]. These results suggest the pivotal importance of clarifying miRNA functions and regulatory mechanisms to formulate new diagnostic and therapeutic strategies.

Increasing evidence suggests that miR-27 functions in several tumor types as either an oncomiR or an anti-oncomiR. Previous studies have demonstrated that the high expression of miR-27b may be one of the critical factors contributing to malignant breast tumors [10]. HPV16 E7 up-regulates miR-27b to promote proliferation and invasion in cervical cancer [11]. In contrast, overexpression of miR-27b reduce murine mesenchymal stem cells migration by directly targeting SDF-1α [12]. MiR-27b targets PPARγ to inhibit growth, tumor progression and inflammatory response in neuroblastoma cells [13]. MiR-27b is a master miRNA capable of enhancing drug sensitivity in cancer cells by targeting CCNG1 to regulate p53 activity [14]. These results suggest that the biological functions of miR-27b-3p are extremely different. Although miR-27b-3p in CRC was originally described as antiproliferation and its reduced expression associated with cancer stem cells and CRC tissues [15], more recent data correlate miR-27b-3p with overall survival of CRC patients [16].

Here, we further studied the roles of miR-27b-3p in CRC. Our results showed that miR-27b-3p alters the cell morphology and intercellular junction pattern of CRC cells. Additionally, miR-27b-3p promotes CRC cell migration and invasion without affecting cell proliferation. Bioinformatics and luciferase reporter assay confirmed that HOXA10 is a direct target of miR-27b-3p. Moreover, inhibition of HOXA10 expression accounted for the downstream effects of miR-27b-3p in CRC. Furthermore, either overexpression of miR-27b-3p or suppression of HOXA10 activates the integrin β1 signaling pathway to mediate the downstream effects of miR-27b-3p. In conclusion, our results reveal the miR-27b-3p/HOXA10 axis and explain why miR-27b-3p promotes migration and invasion in CRC.

## Materials and methods

### Cell lines, miRNA and siRNA

The human colorectal cancer cell lines HCT116, RKO and 293T were purchased from the American Type Culture Collection (ATCC, Manassas, VA, U.S.A.). HCT116 was cultured in RPMI1640 (Gibco Life Technologies, Carlsbad, CA, U.S.A.) with 10% FBS (Gibco). RKO and 293T were cultured in DMEM (Gibco) with 10% FBS. All cells were cultured at 37°C in a 5% CO_2_ humility incubator. The hsa-miR-27b-3p mimics, corresponding negative control and siRNA targeting HOXA10 were synthesized from GenePharma (Suzhou, Jiangsu, China). si-HOXA10-1 sense 5′-CGCAGAACAUCAAAGAAGATT-3′, anti-sense 5′-UCUUCUUUGAUGUUCUGCGTT-3′; si-HOXA10-2 sense 5′-GCAAAGAGUGGUCGGAAGATT-3′, anti-sense 5′-UCUUCCGACCACUCUUUGCTT-3′; negative control siRNA sense 5′-UUCUCCGAACGUGUCACGUTT-3′ anti-sense 5′-ACGUGACACGUUCGGAGAATT-3′.

### Quantitative real-time PCR

TRIzol reagent (Invitrogen, Calsbad, CA, U.S.A.) was used for total cellular RNA isolation. PrimeScript™ RT reagent Kit (TaKaRa, Dalian, Liaoning, China) was used to synthesize cDNA for mRNA expression detection. Mir-X™ miRNA qRT-PCR SYBR® Kit (TaKaRa) was used to reverse transcribe miRNA. The quantitative reverse transcription-PCR (qRT-PCR) was performed using SYBR Premix Ex TaqII (TaKaRa) on the QuantStudio™ 7 Flex System (Thermo Fisher Scientific, Waltham, MA, U.S.A.). miR-27b-3p and mRNA expression levels were normalized to U6 and GAPDH, respectively, and the gene expression fold changes were calculated by relative quantification (2^−ΔΔ*C*t^).

### Cell proliferation assay

The cell counting and EdU incorporation assays were used to evaluate the cell proliferation ability. For EdU cell incorporation assay, HCT116 or RKO cells plating on a coverslip were transfected with miR-27b-3p and NC mimics. Then, 24 h after transfection, EdU cell proliferation assay kit (RiboBio, Guangzhou, Guangdong, China) was applied to quantify DNA replication. Briefly, cells were incubated with 50 µm EdU for 2 h, followed by fixation (4% polyformaldehyde) and permeabilization (0.1% Triton-100). Then, Apollo fluorescent reagent was used for EdU and DAPI for nuclear staining. Apollo fluorescence was excited at 550 nm and emitted at 565 nm and DAPI was excited at 405 nm and emission filters at 460 nm. For cell counting, HCT116 or RKO cells plated on 6-well culture plates were transfected with miR-27b-3p and cultured for the indicated number of days. The cell numbers were read from a TC20 Automated Cell Counter (Bio-Rad, CA, U.S.A.).

### Confocal microscopy

For cytoskeleton assay, HCT116 or RKO cells plated on a coverslip were transfected with miR-27b-3p and NC mimics. Twenty-four hours after transfection, cells were fixed by 4% polyformaldehyde and permeabilized by 0.1% Triton-100. Then, rhodamine phalloidin reagent was applied for filament actin and DAPI for nuclear staining. Rhodamine was excited at 535 nm and emission was received at 585 nm, DAPI was excited at 405 nm and emission filters at 460 nm.

### Western blot assay

Western blot analysis was performed as previously described [17]. Following transfection with miRNA mimics for the indicated times, cells were rinsed twice with PBS and then harvested and lysed with RIPA lysis buffer containing protease inhibitors. Protein samples were separated on SDS-PAGE and transferred to nitrocellulose membrane (Bio-Rad, Hercules, U.S.A.). After blocking with 5% skim milk, membranes were incubated with HOXA10, integrinβ1 (Cell Signaling Technology, Danvers, MA, U.S.A.) and GAPDH (Abcam, Cambridge, U.K.) antibodies accordingly. After secondary antibody incubation, band signal was detected with the use of enhanced chemiluminescence reagent.

### Luciferase reporter assay

A luciferase reporter vector pMir-Report (Ambion, Austin, TX, U.S.A.) was used to generate luciferase reporter construct. The 3′UTR of HOXA10 containing the SacI and MluI restriction enzyme cutting sequences was synthesized. The synthesis DNA was digested with SacI and MluI, followed by insertion into a SacI- and MluI-opened pMir-Report vector to generate the Luc- HOXA10 (WT) construct. To generate the mutant construct Luc-HOXA10 (MUT), the predicted miR-27b-3p binding site was reversed and the construct was generated in a similar way.

Luciferase activity assay were performed using the Dual Luciferase Assay System as described previously. 293T cells were plated for transfection. Lipofectamine 3000 (Invitrogen, Carlsbad, CA, U.S.A.) was used to transfect the indicated firefly luciferase reporters (WT or MUT), corresponding miRNA mimics and Renilla luciferase construct. Then, 24 h after transfection, samples were collected and tested following the guide of Dual-Luciferase Reporter Assay System (Promega, WI, U.S.A.). Luciferase activity of different treatments were normalized to Renilla luciferase activity.

### Statistical analysis

All data that were expressed as the mean ± SD and processed using GraphPad Prinsm7 software were collected from three independent experiments. The difference among the groups was estimated by Student’s *t*-test or one-way ANOVA. A *P* value less than 0.05 was considered to be statistically significant.

## Results

### MiR-27b-3p has no effect on cell proliferation but alters cell morphology in CRC cells

**Figure 1:**
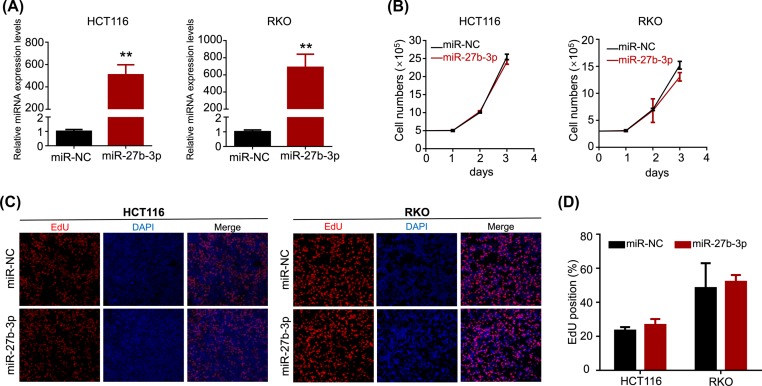
miR-27b-3p showed no effect on CRC cell proliferation (**A**) qRT-PCR was used to verify the expression of miR-27b-3p in cells, 48 h after transfection with 10 nM miRNA mimics or NC mimics. (**B**) Cell proliferation was determined by counting the cells on the first, second and third day after transfection with miR-27b-3p or NC. (**C,D**) Cell DNA synthesis activity was detected by EdU incorporation assay on cells after transfection with miR-27b-3p mimics compared with NC mimics; **P < 0.001.

**Figure 2:**
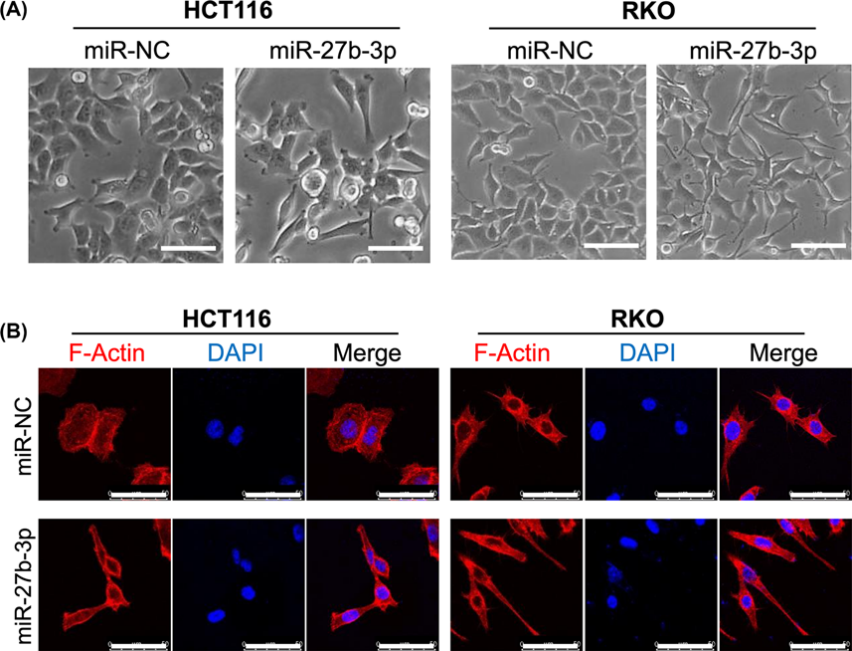
miR-27b-3p changed the morphology and cytoskeleton organization of CRC cells (**A**) Cell morphology was visualized under a phase contrast light microscope after transfection with miR-27b-3p or NC mimics; scale bar = 100 μm. (**B**) A laser confocal microscope was used to visualize the cell. After transfection with miR-27b-3p or NC mimics, phalloidin and DAPI were applied to cells for cytoskeleton (red) and nuclei (blue) staining, respectively; scale bar = 50 μm.

To explore the function of miR-27b-3p in CRC cells, miR-27b-3p was overexpressed in HCT116 and RKO cells by transient transfection with miR-27b-3p and NC mimics. Transfection efficiency was confirmed by RT-PCR. The relative levels of miR-27b-3p expression were significantly higher in the miR-27b-3p transfected cell lines compared with the expression levels of the control cell lines (Figure 1A). It has previously been reported that miR-27b-3p plays a role in inhibiting cell proliferation in colorectal cancer. We therefore performed cell counting to investigate the effect of miR-27b-3p on cell proliferation. However, there was no statistically significant difference in the number of miR-27b-3p-overexpressed HCT116 or RKO cells compared with the control group (Figure 1B). We further tested this result with EdU incorporation assay. Overexpression of miR-27b-3p did not reduce EdU-positive cells in both CRC cell lines (Figure 1C,D). Therefore, we concluded that miR-27b-3p did not affect the proliferation of HCT116 and RKO cells. Interestingly, cell morphology changed 24 h after transfection. The miR-27b- 3p transfected cells had a spindle-shaped morphology and dissociated from each other (Figure 2A). Morphology changes were confirmed by phalloidin staining. The cytoskeleton of miR-27b-3p-overexpressed cells showed reorganization and polarization (Figure 2B).

### MiR-27b-3p promotes migration and invasion in CRC cells

**Figure 3:**
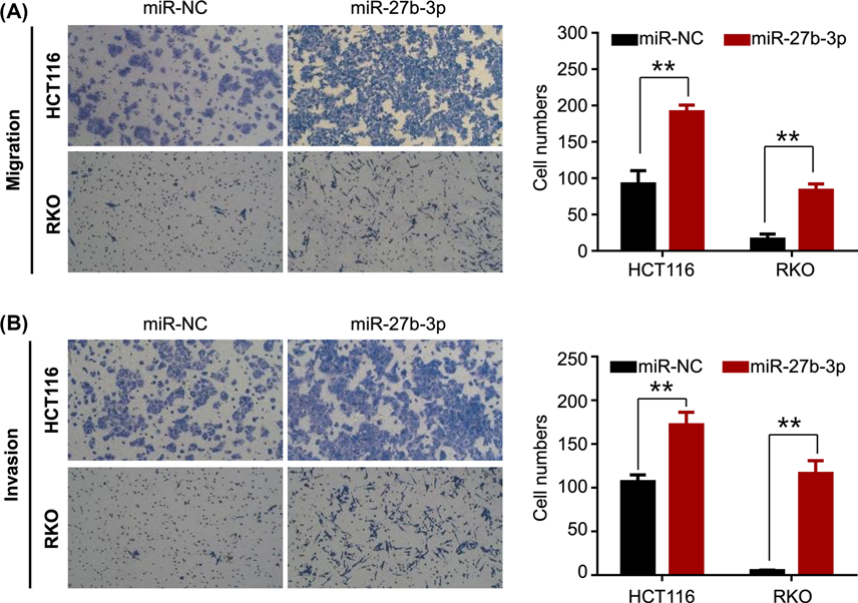
miR-27b-3p significantly promoted CRC cells migration and invasion (**A**) HCT116 and RKO cells transfected with miR-27b-3p or NC mimics were subjected to migration assay. The number of migrated cells was counted and displayed in a histogram on the right. (**B**) HCT116 and RKO cells transfected with miR-27b-3p mimics or control were subjected to the invasion assay. The number of invading cells was counted and shown in a histogram on the right; **P < 0.001.

Loss of cell–cell adhesion may be an important step for tumor cells to acquire invasive and metastatic capability. Considering the elongated and spindle-shaped morphology of miR-27b-3p-overexpressed HCT116 and RKO cells, the effect of the miR-27b-3p on cell motility has been investigated. HCT116 and RKO cells were transfected with miR-27b-3p or NC mimics. The migration and invasion abilities of HCT116 and RKO cells were subsequently detected by Transwell assay. As shown in Figure 3, miR-27b-3p overexpression significantly promoted the migration and invasion of CRC cells.

### MiR-27b-3p directly targets HOXA10 in CRC cells

**Figure 4:**
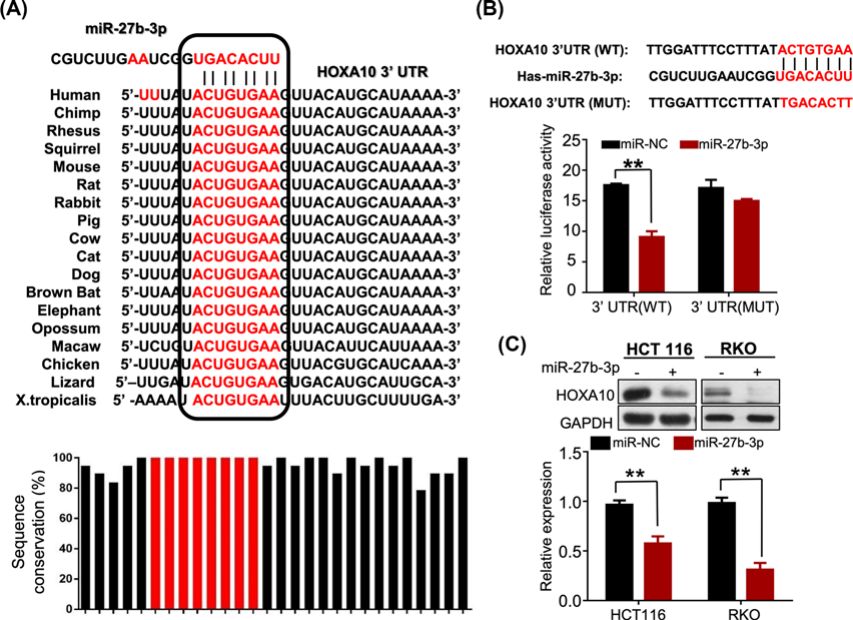
miR-27b-3p directly targets HOXA10 in CRC cells (**A**) The predicted binding of miR-27b-3p with HOXA10 3′UTR across various species. (**B**) A dual luciferase reporter assay was performed to validate miR-27b-3p target HOXA10 intracellularly. Luciferase reporter constructs containing a 3′UTR fragment of HOXA10 were co-transfected with miR-27b-3p or NC mimics in HCT116 cells. A mutated HOXA10 3′UTR fragment was also constructed and performed similarly. Relative firefly luciferase expression was normalized to Renilla luciferase. (**C**) The Western blot was performed to measure the HOXA10 protein levels in CRC cells transfected with miR-27b-3p or NC mimics for 24 h. Protein expression was quantified by band intensity and normalized to GAPDH (shown in the lower panel); **P < 0.001.

Bioinformatics analysis was used to identify potentail targets of miR-27b-3p, which may affect the cell ability of migration and invasion The binding site of miR-27b-3p was estimated to be within the 3′UTR region of HOXA10 by the TargetScanHuman 7.2 database [18]. Examination of the target sequences indicated that the miR-27b-3p target sites were highly conserved among different species. As shown in Figure 4A, the seed regions that critical for miR-27b-3p binding were 100% homologous to HOXA10 in sequences from all species. To validate whether HOXA10 was the target of miR-27b-3p, we generated that the WT or mutated 3′ UTRs of HOXA10 gene contained binding sequences for miR- 27b-3p, respectively, into luciferase reporter constructs, followed by transfection with miR-27b-3p mimics or control oligo and performed luciferase assays. The results showed that miR-27b-3p significantly reduced the relative luciferase activity in Luc-HOXA10-WT transfected cells. Mutation in the predicted miR-27b-3p binding site counteracted the inhibition of miR-27b-3p on HOXA10 (Figure 4B). To determine whether miR-27b-3p affected HOXA10 expression in the CRC cells, HOXA10 expression level was evaluated in HCT116 and RKO cells following transfection with miR-27b-3p mimics or control oligo. Transfection with miR-27b-3p mimics resulted in a significant decrease in expression of HOXA10 protein (Figure 4C).

### Overexpression of miR-27b-3p increases integrin β1 expression

**Figure 5:**
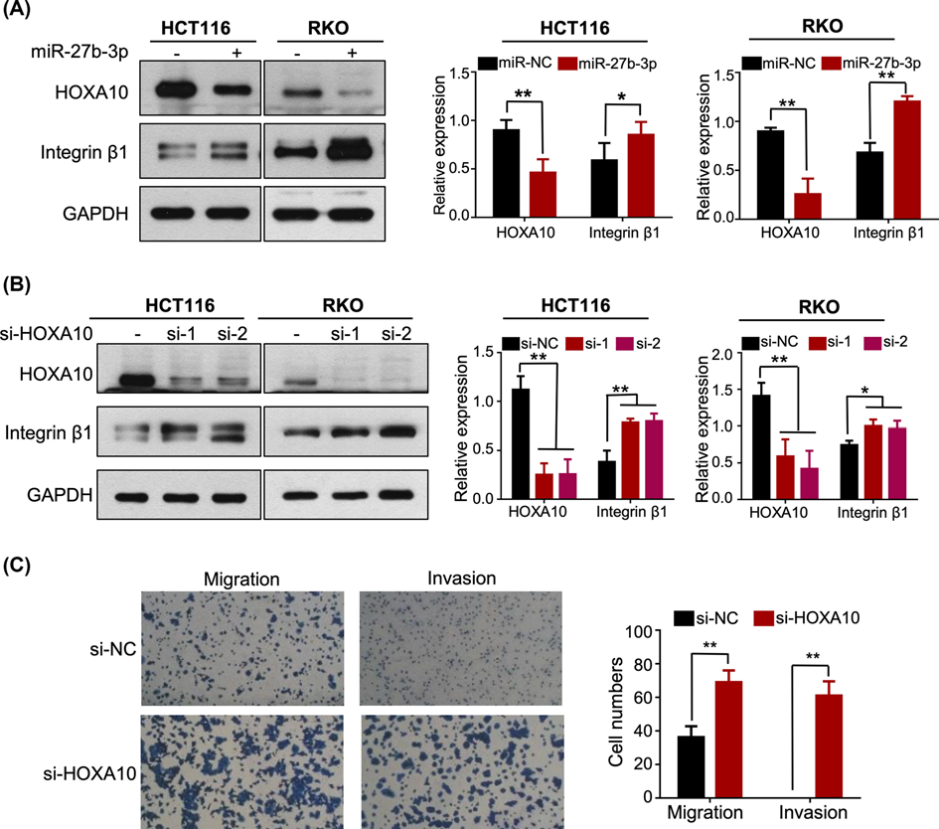
miR-27b-3p/HOXA10 axis promotes the activation of the integrin β1 pathway (**A**) Western blot for HOXA10 and integrin β1, in HCT116 and RKO cells transfected with miR- 27b-3p or NC mimics. Protein expression was quantified by band intensity and normalized to GAPDH (shown in the lower panel). (**B**) Western blot for HOXA10 and integrin β1 in HCT116 and RKO cells transfected with targeted HOXA10 or NC siRNA. Protein expression was quantified by band intensity and normalized to GAPDH (shown in the lower panel). (**C**) HCT116 cells transfected with HOXA10 targeted siRNA or NC siRNA were subjected to migration and invasion assay. The number of migrating or invading cells was counted and shown in a histogram on the right; *P < 0.05, **P < 0.001.

Due to the overexpression of miR-27b-3p, we have previously observed that cytoskeleton changes with the dysregulation of the intercellular junction pattern, which prompted us to further investigate the association of miR-27b-3p with cell adhesion pathway. It has been reported that the integrin β1 signaling pathway plays a critical role in cell morphology, migration and invasion in CRC cells [19, 20]. To examine whether the migration and invasion promoted by miR-27b-3p involved the integrin β1 signaling pathway, expression of integrin β1 was determined by western blot. As we expected, overexpression of miR-27b-3p or suppression of HOXA10 significantly increased the expression of integrin β1 (Figure 5A,B). Moreover, depletion of HOXA10 in HCT116 also promoted cell migration and invasion (Figure 5C). These data also demonstrated that the integrin β1 pathway is a downstream functional regulator of miR-27b-3p.

## Discussion

MiR-27b-3p has been reported to be involved in multiple biological pathways that can affect tumor progression and metastasis [10, 15, 21–25]. Therefore, it is crucial to delineate a comprehensive overview of miR-27b-3p. However, the biological basis for the supposed prognostic impact of miR-27b-3p is not yet clarified, because of conflicting results. For example, miR-27b-3p is primarily reported to be an oncogene in breast cancer. Once miR-27b is up-regulated in breast cancer, it causes down-regulation of Nischarin and activation of the NFκB signal pathway, followed by further increase in miR-27b transcription [10]. However, another group reported that miR-27b suppresses breast cancer cells proliferation, colony formation, and promotes chemosensitivity and paclitaxel-induced apoptosis [26]. These results suggest that miR-27b-3p may have a variety of cellular functions in different cells. Moreover, the biological basis for miR- 27b-3p has not yet been clarified.

Compared with breast cancer, the studies in CRC are more consistent, two groups reported that miR-27b-3p was decreased in most CRC tissues, and overexpression miR-27b-3p inhibit the proliferation, migration and invasion in CRC cells. To more comprehensively study its role in CRC, an additional colorectal cancer cell line, RKO, was included in our experiments. Inconsistently, our data did not demonstrate a significant inhibition of proliferation in cells exhibiting miR-27b-3p overexpression and the aforementioned EdU experiment result further confirmed our observation.

In terms of cell motility, we discovered that overexpression of miR-27b-3p dramatically promotes migration and invasion in two representative colorectal cancer cell lines, HCT116 and RKO. This increased motility combined with changes in cell morphology that we observed under a phase contrast light microscopy, revealing that overexpression of miR-27b-3p induces an elongated and spindle-shaped phenotype. Furthermore, miR-27b-3p-induced cytoskeleton reorganization can be clearly observed under the laser confocal microscopy. However, it significantly promotes the migration and invasion of CRC cells. This suggests that the functional diversity of miR-27b-3p exists not only in different types of tumors, but also in the same type of tumor. Our findings are consistent with previous observation that miR-27b-3p promoted migration and invasion in breast cancer [10]. However, despite showing the similar biological effects, molecular mechanisms underlying miR-27b-3p activity appears to be different, which further confirming the importance of the cellular context in the activity of miR-27b-3p.

By bioinformatics analysis (TargetScan 7.2), HOXA10 is the target gene of miR-27b-3p, which is confirmed by luciferase assay and western blot. Homeobox (HOX) genes are well known for their critical role during normal embryogenesis, in the patterning and development of the embryonic body plan. They have also been found to be important in oncogenesis [27,28]. HOXA10, a member of HOX genes, is involved in regulating differentiation and progression in several cancer types [29–32]. Other members in the HOX gene family have also been shown to play various roles in CRC. HOXA5, a paralog of HOXA10, has previously been reported to be down-regulated in colon cancer cells and its re-expression induces loss of the cancer stem cell phenotype, thereby preventing tumor progression and metastasis [33]. Similarly, knockdown of HOXA10 in CRC cells promoted cell migration and invasion. These results indicate that miR-27b-3p promotes cell migration and invasion, partially by inhibiting HOXA10.

Integrin β1 is one of the most important members of integrin family, which regulates a diverse array of cellular functions to promote cytoskeleton remodeling and alter cell morphology, motility and invasiveness [19,20]. In the present study, we revealed miR-27b-3p increases the integrin β1 pathway by targeting HOXA10. Further study showed that si-HOXA10 promotes activation of the signaling pathways. Therefore, we hypothesized that miR-27b-3p activates the integrin β1 signaling pathway by targeting HOXA10. The unknown details of this activation mechanism require further investigation.

## Conclusion

**Figure 6:**
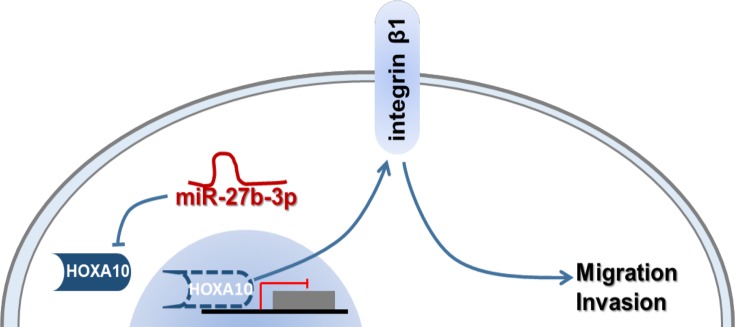
A schematic model of the mechanism demonstrating how miR-27b-3p promotes colorectal cancer cell migration and invasion

Taken together, the present study provides evidence that miR-27b-3p represses HOXA10 directly and promotes cell motility via integrin β1 pathway (Figure 6). This finding provides a mechanism to explain how miR-27b-3p promotes migration and invasion in CRC cells.

## Abbreviations

DAPI: 4′,6-diamidino-2-phenylindole
EdU: 5-ethynyl-2′- deoxyuridine
GAPDH: glyceraldehyde-3-phosphate dehydrogenase
MUT: mutation
NC: negative control
qRT-PCR: quantitative real-time polymerase chain reaction
siRNA: small interfering RNA
UTR: untranslated region
WT: wild-type
